# Notes on the Use of Ether in Obstetrics

**Published:** 1884-03

**Authors:** Casey Albert Wood

**Affiliations:** Professor of Pathology, Medical Faculty, University of Bishops College, Physician to the Western Hospital


### NOTES ON THE USE OF ETHER IN OBSTETRICS.*

BY CASEY ALBERT WOOD, C.M., M.D.,

*Professor of Pathology, Medical Faculty, University of Bishops College, Physician to the Western Hospital.*

[Canada Medical Record.]

Until about a year ago it was my invariable rule to employ chloroform in midwifery, not only for the alleviation of pain during the first and second stages of labor, but also for the performance of any of the operations incident to obstetrics. Since that time I have considered it advisable to modify my practice in some respects, and to substitute ether; and in this paper I propose shortly to give my reasons therefor, and to ask of members of this Society, whose larger experience warrants their speaking with authority, their opinions upon the subject.

The only apology I have to offer for the assumption that it is possible to come to any conclusions of value in a small number of cases —twenty-six in all—is that attendance upon cases of midwifery, where it is necessary to employ anæsthetics, gives one ample opportunity to study their effects in each instance; for the moderately careful observer, who stays up half the night in the endeavor to relieve a parturient female, is likely to have sufficient chances of watching the progress of events and the extent to which they are influenced by the administration of remedies. In those cases where relief is called for in the first stage of labor, examples of which are most commonly found among primiparæ, where a slowly dilating or rigid os is represented by sharp pains, nervous excitability, inability to sleep, and after a time by exhaustion, I have usually been able to succeed in quieting the patient and obtaining rest by giving her a full dose of chloral; or, if that fail, by administering a few doses of morphia. After a few hours of quiet, dilatation proceeds more quickly, and by the time the effects of the opiate have passed away the labor has progressed to the second stage. In the beginning of October, however, I had a patient who refused to exhibit this satisfactory phase of affairs. She was about to be confined of her second child; had been delivered of her first by the use of forceps after a prolonged labor, and was in great dread of a second ordeal. In addition to her nervousness the membranes ruptured after the os had dilated to the extent of a ten-cent piece, revealing

*Read befo th Medico-Chirurgical Society of Montreal. December 28th, 1883.

an occipito posterior presentation. The pains were not very severe or very frequent, but they appeared to exhaust the patient, who insisted upon my giving her chloroform. I had a bottle of Squibbs' ether with me, and I proceeded to administer the anæsthetic in the usual intermittent way. I noticed, however, that the effect produced corresponded mainly to the *intervals* between the pains, and while it quieted her and gave her some sleep it did not "take the edge" off the pains as chloroform had previously done in my hands. As the affair progressed she seemed to gain courage and strength, rotation was accomplished, and the second stage was passed without the necessity of instrumental aid. As I walked home that night I felt that in that particular instance it would have served my purpose better to have given chloroform. A short time ago I attended a young woman, primipara, who had a long, tedious labor, and to whom I began the administration of ether when the head, presenting in the first position, had proceeded fairly into the second stage. She had been suffering from hemorrhoids for several days previously and was tired out before labor began. I gave her Squibbs' ether during and for a moment before the advent of each pain for over two hours. At the end of that time I endeavored, with the aid of the nurse, to apply forceps, but, owing to the difficulty with which she was brought under the influence of the anæsthetic, I was obliged to send for my friend, Dr. Gaherty. With his aid she was safely delivered, and recovered rapidly and nicely from the ether and from the effects of the long labor. I questioned her closely, and she declared that she felt little or no pain from the time of my first administration of the anæsthetic until I determined to apply forceps. This case is a fair sample of my experience in ether administration during the second stage of labor. Where the pains are sufficiently severe, and the condition of the patient such as to warrant it, ether —good ether I mean—seems to me to furnish all the satisfactory results, both as regards its present and remote effects, that chloroform does, provided you give it slightly in anticipation of the pains. I have had a number of cases of severe hemorrhage following the administration of chloroform given to produce complete anæsthesia while the forceps were used. So much so has this been the case, in my experience, that I have always looked out for at least a smart temporary postpartum bleeding, and usually found it. As far as I can judge from the small number of cases where ether was given, I do not think such hemorrhage has been as frequent or as troublesome. * * *

Without multiplying the record of cases, valuable only as bearing on the question at issue, I would recount the relative merits, *mea sententia*, of chloroform and ether in obstetrics something as following: (1) Owing to the agreeable odor, early effects, and perfect safety of chloroform as an anodyne agent, it is, without the least doubt in my mind, the agent best suited to alleviate the pain and calm the nervous irritability incident to the first stage of labor. (2) This statement is generally true of the expulsive period, where complete abolition of pain is not the object of the administration. (3) When, however, complete anæsthesia is required, as we find it necessary during the delivery of the child, and for the performance of operations following or preceding delivery, then it seems to me that chloroform largely loses its character as the obstetrical anæsthetic *par excellence.*

If it be acknowledged that considerations of safety must give way, in general practice, to greater conveniences of administration, etc, then, too, in the operations of midwifery, ether must supplant chloroform. If it can be shown that there is anything about the parturient woman which renders her less susceptible to danger during chloroform inhalation which does not equally apply to ether, then the force of this argument is much lessened. So far as I know, this peculiar immunity does not exist. We know that it is in the practice of midwifery that the use of anæsthetics is considered least dangerous. By a process of natural selection, as it were, we then have women in the prime of life generally free from disease, with all their nutritive functions in good order—they naturally form the best class of patients to which *any* anæsthetic could be given—and this aside from the theories commonly put forward to explain such immunity from accident, such as increased cardiac development, the physiological cerebral congestion guarding against syncope, brought about by the effects of the uterus to expel its contents, and so on.

Other considerations may serve to modify these conclusions in the minds of practitioners, and the first one is the inflammable nature of ether and its explosive quality when mixed with a certain percentage of atmospheric air. The kindling point of ethereal vapor and of its dilutions with oxygin is low, and when either of them comes in contact with flame an explosion is sure to follow. As the operations of the obstetrician occur frequently at night time this is a serious objection. The difficulty can be greatly overcome by a little care in preventing the near approach of flame to the inhaler or ether bottle, by thorough ventilation of the room, and by the exclusive use of covered lights. A common lamp is a very crude safety lamp, but it is a great improvement upon such naked flames as a gas jet, wax candle, or other unprotected light. I have never had an accident from an ether explosion. I think the danger could be nullified by the use of a modified Davy lamp.

In my experience vomiting is as of frequent occurrence after the use of ether in midwifery as of chloroform, and I do not think it occurs very often in either case. I think it will be generally admitted that, in view of the danger from post-partum hemorrhage, danger to the child and inherent danger to the mother, it would be more advisable to give ether, for its general anæsthetic effects for a long period, say an hour or longer, than to give chloroform for a corresponding period. Now it often happens that one is obliged to administer, in midwifery, an anæsthetic for a much longer time than was first anticipated, in which case it would be at least advisable to substitute ether for chloroform when a commencement had been made with the latter. I assisted, last May, the President of this Society to deliver a woman whose labor was complicated by a labial hæmatocele which had burst and caused considerable hemorrhage. In this instance I am sure that, remembering the length of time she was under ether, about an hour and a half, the ease with which she was kept under its influence, the confidence with which its administration was left for a large portion of the time to the nurse—all these made me feel that ether was the anæsthetic for that particular case. I have here to refer to the matter I have just spoken of—the confidence with which, in view of its greater safety, the administration of ether can be given over to the nurse or to anyone whom the exigencies of the case have left in possession of her faculties.

In country places this rule applies with greater force than it does to city practice; but it often happens that even in the city it is not convenient, desirable or necessary to call in a brother practitioner. In such cases, it seems to me that ether possesses considerable advantage over chloroform. Finally, in labors fatal to the mother, where an anæsthetic has been employed for any length of time—and I have as yet fortunately had no experience of such cases—it may be a relevant question to ask, would it not be a satisfaction to know that ether had been given and not chloroform?

